# GWEP TAG-TEAM Response: COVID-19 Health Disparities in Orange County, California

**DOI:** 10.1093/geroni/igab046.343

**Published:** 2021-12-17

**Authors:** Jung-Ah Lee, Julie Rousseau, Neika Saville, Sonia Sehgal, Lisa Gibbs

**Affiliations:** 1 University of California, Irvine, Irvine, California, United States; 2 University of California, Irvine, Orange, California, United States; 3 Division of Geriatric Medicine and Gerontology, Orange, California, United States; 4 UC Irvine Health, UC Irvine Health, California, United States

## Abstract

Health disparities follow zip codes, and in Orange County, CA, both COVID-19 cases and deaths are highly concentrated in our diverse geriatric populations in Santa Ana (44,075) and Anaheim (40,984) where our two UCI Federally Qualified Health Centers (FQHCs) are located, and Garden Grove (16,174) and Buena Park (7,581), where University of California Irvine (UCI) TAG-TEAM GWEP community partner FQHCs are located. Collectively, our FQHCs serve diverse populations, with 83-88% of patients identifying as Hispanic/Latino or Asian. As we support these clinics in becoming Age-Friendly Health Systems, UCI’s GWEP pivoted to provide COVID-19 education in the form of multi-lingual materials and videos available in Spanish, Vietnamese, Korean, Mandarin, and Farsi. Additionally, through our Age-Friendly Geriatrics Tele-ECHO Series we are working to build Mental Health care competencies among these FQHC providers since the pandemic morbidity and mortality disparities have resulted in a profound mental health crisis in our communities.

